# Potential impact of functional biomolecules-enriched foods on human health: A randomized controlled clinical trial

**DOI:** 10.7150/ijms.70435

**Published:** 2022-03-06

**Authors:** Marco Tatullo, Benedetta Marrelli, Caterina Benincasa, Elisabetta Aiello, Massimiliano Amantea, Stefano Gentile, Noemi Leonardi, Maria Luisa Balestrieri, Giuseppe Campanile

**Affiliations:** 1 Department of Basic Medical Sciences, Neurosciences and Sense Organs, P.ce G. Cesare 11, University of Bari Aldo Moro, 70124 Bari, Italy;; 2 Marrelli Health - Tecnologica Research Institute, Biomedical Section, Street E. Fermi, 88900 Crotone, Italy;; 3 Department of Precision Medicine, University of Campania Luigi Vanvitelli, Via L. De Crecchio 7, 80138 Naples, Italy;; 4 Department of Veterinary Medicine and Animal Production, University of Naples Federico II, 80137 Naples, Italy.

**Keywords:** buffalo milk, betaine, healthcare

## Abstract

Naturally occurring milk compounds have recently been investigated for their health-promoting properties; in fact, their anti-microbial, immuno-modulatory, antioxidant and anti-thrombotic activities, have increasingly gained interest within the scientific community. We have reported a translational, randomized, controlled clinical trial (RCT) on human subjects with a moderate to high cardiovascular risk, and a body mass index (BMI) >25.1 kg/m2, to evaluate the clinical impact of biomolecules-enriched Mediterranean Buffalo (*Bubalus bubalis*) milk and its derived dairy foods, produced with innovative breeding techniques. The experimental arm involved patients that followed a diet including the above-described products (treated group; n= 11); the control arm was based on a diet including cow milk and its dairy products (control group; n= 9). The results of this study have been statistically evaluated, pointing out a specific significance related to the comparative analysis of the blood pressure among the 2 arms; in fact, this value showed a significant improvement in an extremely short experimental time. Nevertheless, this study also reported not-significant results that were indicative of an interesting and promising tendency in modulating specific diet-depending haematological and biomedical values. In conclusion, this RCT has assessed that the foods derived from buffalo milk naturally enriched with biomolecules, was able to improve the overall blood glucose levels, the BMI and the body weight. These preliminary results are suitable for the design of future strategies in the prevention of cardiometabolic diseases, thus improving the overall quality of life and the policies of healthcare management.

## 1. Introduction

### 1.1. Background

Functional foods are considered as healthy supplements correctly added to a balanced diet and an active lifestyle.

In the last years, several foods have been reported to be a rich source of bioactive molecules, called “nutraceuticals”; these molecules are able to modulate several biological processes, and to improve the clinical effects of numerous pathologies. Nutraceuticals are typically bioactive proteins and peptides with a high-degree oral bioavailability; importantly, such molecules may retain antihypertensive, anti-inflammatory, and immunomodulatory properties, and may decrease the incidence of metabolic disorders and some types of cancer 1.

Buffalo milk is considered a healthy food, because of its low concentration of cholesterol, and high amount of unsaturated fatty acids 2.

In high-incoming Countries, the increased incidence of dysmetabolic diseases is closely related to wrong lifestyle and the high consumption of junk foods: in this landscape, the food industry is working to enrich common products with antioxidants, substances capable of reducing the oxidative stress 3.

Casein, Selenium, Zinc, Glutathione peroxidase, Superoxide dismutase, Vitamin E and C, and Beta-Carotene are antioxidant molecules that have been found in milk 4. Buffalo milk have recently been investigated because of their extraordinary concentration of proteins, minerals, vitamins, and antioxidants 5: studies based on gas-chromatographic analyses have revealed that buffalo milk, compared to cow's milk, contains more omega-3 and CLA (Conjugated Linoleic Acid), which are essential fatty acids impossible to be synthesized by human body autonomously.

Among the studies on buffaloes' diet, a pivotal role has been played by protocols based on the integration of green forage: in fact, the experimental diet based on green forage has been demonstrated to increase polyunsaturated fatty acids and improve omega-3/omega-6 ratio in buffalo milk 6. Importantly, among the omega-3 fatty acids, α-linoleic fatty acid (LNA, C 18: 3 omega-3) retains an important role in reducing triglycerides, thus preventing human thrombosis by means of a massive inhibition of thromboxane A2 6,7.

The polyunsaturated acids (PUFA) n-6 and n-3 are nutraceutical compounds positively acting on coronary heart disease (CHD) and on some tumour types 8. The PUFAs obtained from buffalo milk seems to have immunosuppressive effects on such diseases characterized by endothelial inflammation and hyperimmune response 9-11.

On the other hand, buffalo milk is a functional food with cholesterol levels lower than cow milk 12, and high levels of proteins with important nutraceutical effects. Also, buffalo milk is rich in important mineral salts 13. Recently, researchers have specifically investigated on buffalo Casein: this protein contains low amount of sialic acid (2. 0mg.g-1 casein), hexose (2.5mg) and hexosamine (1.8mg); on the other hand, some peptides, identified by RP - HPLC technique, are able to induce the proliferation of osteoblasts, leading to a better bone formation 14, 15. In conclusions, buffalo milk has important properties that highly impact the human health: the natural presence of strategic proteins, antioxidant compounds and other bioactive molecules make buffalo milk a functional food worthy of further investigations.

### 1.2. Functional molecules: the role of Betaines

Betaines are natural compounds extracted from several vegetables, such as quinoa, spinaches, and broccoli. Chemical structure of Betaines is based on a methyl derivative of glycine, and they are metabolized to dimethylglycine and sarcosine in the liver 16-18. Many studies on animal models have suggested that betaines, administered with dosages ranging from 500 to 900 mg / day, could promote the reduction of general amount of abdominal fat, basically through a balanced increase of lipolysis, and the contemporary inhibition of lipogenesis. The pathway involved in this clinical outcome is regulated through the stimulation of endocrine IGF-1 release, able to increase the creatinine synthesis 19.

Betaines are functional molecules with impact also on liver function and detoxification, as they are considered "methyl donors" like some B vitamins.

Betaines also increase the liver ability to metabolize the local hepatotoxins before they can induce damages to the digestive tract 20.

Besides a high content of natural antioxidants such as tocopherols and peroxidases, buffalo milk is also rich in Betaines, including glycine-betaine and δ-valerobetaine (δVB) 16, 17. δVB showed significant efficacy in reducing reactive oxygen species, lipid peroxidation and cytokine release during high glucose (HG) treatment. δVB also reduced the HG-activated inflammatory signal by modulating several Sirtuins, such as sirtuin-1 (SIRT1), sirtuin-6 (SIRT6), and the transcription factor nuclear factor kappa-light-chain-enhancer of activated B cells (NF-kB) 21. The regulation of SIRT1 and SIRT6 also has antioxidant effects, impacting the main metabolic pathways that lead to several chronic-degenerative diseases, such as type 2 diabetes, osteoporosis, hypertension, cancer, cardiovascular disease 22-25. In conclusion, δVB showed significant efficacy in reducing oxidative stress and local inflammation, suggesting a potential role of this betaine as a new dietary compound retaining beneficial properties for health 12.

Buffalo milk is naturally rich in activators of δ-valerobetaine, which is biosynthesized from trimethyl-lysine, a metabolite involved in the biosynthesis of carnitine 26, 27. Also, buffalo milk is used to product dairy products greatly appreciated for their nutritional properties. The peptides identified in buffalo yogurt, mozzarella, and ricotta belong to the class of Caseins (α s1-, β-, k-CN) and whey proteins (α-LA, β-LG): the studies on such foods have clearly showed antihypertensive, immunemodulatory, antimicrobial, antidiabetic, antitumor and antioxidant activities 28-32.

### 1.3. Functional foods: the role of buffalo milk

The research on functional foods is a field of growing interest, developing novel and traditional foods with several interesting nutraceutical properties 33. Generically, the terms “superfood” or “functional food” mean a food able to achieve changes and improvements within the human body; such foods have been demonstrated to play a strategic role in the prevention of degenerative and inflammatory diseases, thus promoting the health of general population. In the last years, functional foods have been even more included in a balanced diet, to strengthen the prevention of inflammatory diseases and to promote a healthy lifestyle of general population 34.

A functional food can be also obtained through biotechnological treatments aimed to enrich or deprive natural foods of selected compounds. In fact, bioactive compounds are well known, and they are usually plant-derived substances, such as flavonoids, phytosterols and isoflavonoids, or animal-derived molecules, such as omega-3 fatty acids 35.

Buffalo milk has general characteristics very similar to cow milk, apart for the higher level of fats and proteins. Moreover, some strains of “lactobacilli” can be more represented in buffalo milk than in cow milk 36. Compounds like calcium, phosphorus, riboflavin, and vitamin B12, are also more abundant in buffalo milk, where it can be found an extraordinary amount of vitamin D 37.

Several studies have shown that buffalo mozzarella and cow mozzarella differ significantly in their chemical composition and microbial population. Already in 2016, an article published in the *Food Chemistry journal* highlighted the microbiological and metabolomic profiles of buffalo mozzarella compared to other ones; in particular, buffalo mozzarella has a greater microbial diversity, higher levels of threonine, serine, and valine, and lower levels of urea 38-39. Cow mozzarella, on the other hand, has a higher concentration of *Streptococcus thermophilus,* and higher levels of complex sugars 40.

### 1.4. Aim of the work

Milk and dairy products are among the main sources of carnitine: interestingly, a low availability of carnitine does not allow the passage of fatty acids into the mitochondria and therefore causes their consequent accumulation in the cytoplasm of the cells 30-32. Furthermore, according to Salzano et al., the buffalo milk has several functional metabolites, produced on the basis of the buffalos breeding technique 33. In detail, the amount of bioactive functional compounds found in buffalo milk seems to be depending on the buffalo's well-being, which directly impacts the oxidative stress levels observed in the animals 33. In this landscape, the development of new feeding techniques, such as a diet based on green forage, may increase the concentration in the milk of L-carnitine, acetyl-L-carnitine, propionyl-L-carnitine and δ-valerobetaine. Furthermore, the green feed is able to increase the antioxidant molecules found in buffalo milk 2.

Recent research trends have pushed to improve buffalo milk, making it a naturally obtained superfood. The experimental conditions to improve buffalo milk have been evaluated in a pilot study based on an innovative farming technique investigated at Le Verdi Praterie, a Calabrian-based Company that has arranged increases spaces inside the buffaloes' paddocks, and has administered green fodder, ensuring an optimal general metabolic condition to the buffaloes involved in this study.

The aim of this article is to describe the main outcomes obtained from a randomized, controlled clinical trial (RCT) conducted on subjects with moderate to high cardiovascular risk and body mass index (BMI) >25.1 kg/m2, to evaluate the impact of Mediterranean Buffalo (*Bubalus bubalis*) milk and dairy foods produced with breeding techniques capable of increasing the content of fatty acids, acylcarnitine, and betaines.

## 2. Results

The study has first assessed the main nutritional characteristics of the milks used for this pilot study: specifically, bovine milk, buffalo milk, and a novel buffalo milk obtained through a feeding and housing experimental protocol (experimental milk) (Table 1).

Furthermore, the main nutritional characteristics of a specific milk derivative (mozzarella cheese) have been also investigated and compared (**Table 2**).

The subjects selected and allocated in each of 2 experimental arms (Treated and Control) were investigated at baseline (T_0_) with clinical and haematological examinations, then repeated at T_2_, after 3 months, as reported in **Table 3**.

The patients involved within the study have shown significant percent variations of body mass index (BMI) (**Table 4**, P < 0.05) between control and treated groups.

**Figure 1:**
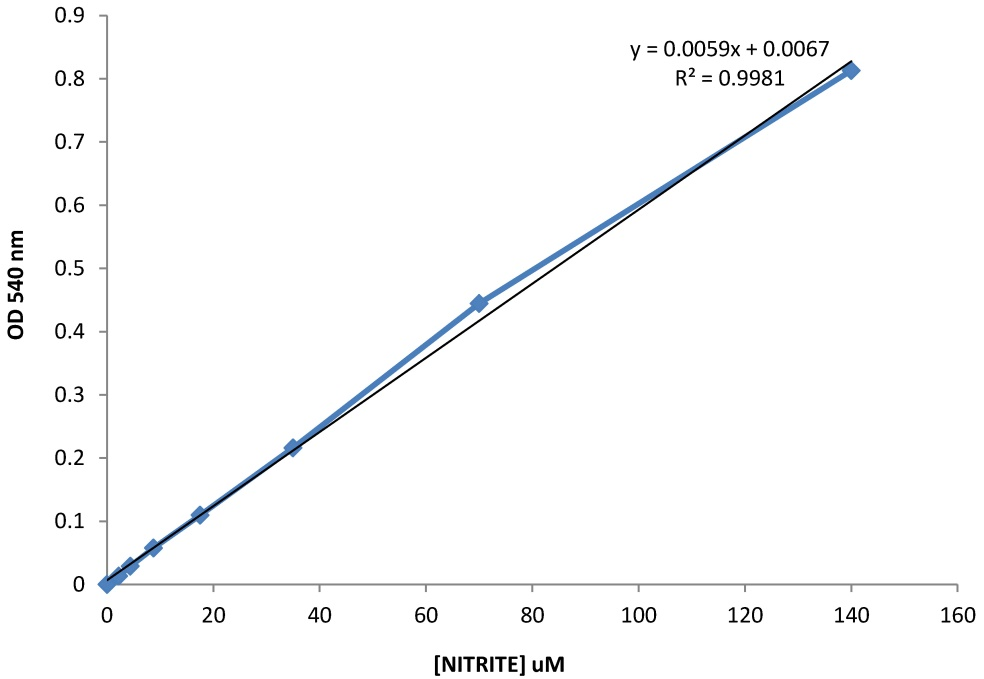
Evaluation of intracellular nitrite concentration, a marker for the analysis of oxidative stress.

The results obtained from the analysis of intracellular nitrite concentration, a marker for the analysis of oxidative stress, revealed that in both the groups the values were tendentially decreased from T_0_ to T_2_. Such tendency suggests that the diet based on buffalo products, enriched with betaines, may have impact in reducing the intracellular oxidative stress (**Figure 1**). Nevertheless, in the control group, the variation from T_0_ to T_2_ does not appear to be homogeneous: in fact, some patients reported values from time T_0_ to T_2_ increased, differently from other patients within the same group, where these parameters seem to follow a different and opposite trend.

The results of this pilot study have been statistically evaluated, paying specific attention to the blood pressure variation, despite we have carried out this study in a very short experimental time. Nevertheless, the other results also may indicate an interesting and promising tendency in modulating specific diet-depending haematological and biomedical values. The impact of dietary enriched with these superfoods may be an innovative strategy to treat and prevent the pathologies impacting on quality of life and overall health.

Finally, we must consider this pilot study as a promising approach to a novel concept of functional diet: in fact, despite the data have not a robust significance among the two groups, we can see a clear tendency in blood pressure and in other important values, such as glycemia and BMI, body weight and lipid values, which could be influenced by a specific diet, specifically when it is rich in functional molecules.

## 3. Discussion

In this work, we evaluated the nutraceutical activity of experimental buffalo milk and dairy products, produced following an innovative method, able to increase content of the biomolecules of such foods. The experimental diet has been administered to subjects suffering from cardiovascular diseases linked to an impairment of specific clinical and hematological factors.

Buffalo milk is a food rich in fatty acids: the importance of fatty acids is strategic in several biological pathways. Interestingly, the arachidonic acid has a structural and functional role in cell and subcellular membranes; moreover, it is also a precursor of prostaglandins, thromboxane and leukotrienes, resulting involved in several inflammatory conditions, such as the cardiovascular diseases 41. On the other hand, fatty acids have been demonstrated to induce changes in several metabolic pathways: more in detail, they can promote the reduction of blood triglycerides and the reduction of the atherosclerotic processes affecting the main arteries, thanks to the inhibition of the synthesis of cytokines and interleukins. Finally, fatty acids have an important role in the prevention of vascular thrombosis, mainly thanks to the inhibition of the synthesis of thromboxane that causes a reduction of the inflammatory response 42-43.

However, researchers say excess amounts of omega-3 fatty acids can impair immune function and even lead to a dysfunctional immune response or viral or bacterial infection 44. A study d by Fenton J et al., reported that mice fed large amounts of omega-3 fatty acids in the diet showed an increased risk of colitis and immune alteration 45. Therefore, in general, in an ideal diet the ratio between Omega-6, whose intake determines the production of molecules that stimulate inflammation (pro-inflammatory effect), and Omega- 3 with an anti-inflammatory effect should be proportionate (1-2: 1), so as to ensure a certain balance between inflammatory response and anti-inflammatory action. However, Western eating habits have shifted this balance strongly in favor of Omega-6s and, consequently, of their pro-inflammatory action, with negative effects on health 46.

An excess of omega 6, especially linoleic acid, is harmful for the metabolism of omega 3. According to molecular mechanisms, both are processed in the cells thanks to the effect of the same enzymes. Therefore, the excess of omega 6 tends to "occupy" all these biological catalysts, compromising the metabolism of omega 3 (for example, it reduces the production of eicosapentaenoic acid EPA and docosahexaenoic DHA) 47. The analyses functional molecules, in particular, the content of betaines, phospholipid precursors, carnitine and short-chain acyl-carnitines in milk, milk derivatives, subsequently administered in subjects of group treated, showed interesting results. In fact, the improved LVP farm well-being led to an improvement in the chemical and nutritional characteristics of milk showing, higher concentrations of carnitine, acetyl-l-carnitine, propionyl-l-carnitine, glycine betaine, and δ-valerobetaine 33. The betaine recently investigated biomolecules in milk, δ-valerobetaine, is a constitutive milk metabolite with antioxidant and anti-inflammatory activities 1,16,22. In this study, the impact of betaines on vascular and metabolic dysfunctions could result in cardioprotective and antidiabetic action, as demonstrated by the reduction in blood glucose levels in the patients enrolled in our study, specifically, a percent variance equal to -1.56 ± 1.62 for the control group and -3.66 ± 3.23 for the treated group was calculated. Furthermore, δ-valerobetaine displayed antiproliferative effects in human colorectal cancer cells and oral squamous cells, with a highest potency displayed when acting in synergism with γ-butyrobetaine 1-48. Results from our pilot study suggest that buffalo milk and dairy products enriched in carnitine, short-chain acyl carnitines and δ-valerobetaine can be considered precious aid against the onset of metabolic and inflammatory diseases. The observed improvement in blood glucose levels in subjects, the slight lowering in blood pressure and the decrease in BMI (percent variance equal to 18.70 ± 3.55 for the control group and 4.13 ± 1.77 for the treated group) and body weight ( (percent variance equal to 1,78 ± 11,00 for the control group and 4,20 ± 1,54 for the treated group) in most of the enrolled patients, along with an increase in the protective effects against oxidative stress, represent the first translation into clinical evidence of the *in vitro* antioxidant and anti-inflammatory activity of functional biomolecules-enriched buffalo milk. Undoubtedly, further detailed analyses in a large number of randomized patients are required to sustain the potential of buffalo milk and dairy products as components of a balanced dietary pattern in the setting of preventive strategies to reduce the risk of cardiometabolic diseases. Moreover, the management of the large data could be more effective with AI-based programs of data-mining and data fusion, so to digitalize and anonymize the clinical information 49.

## 4. Materials and Methods

### 4.1. Milk sampling and composition

This study aimed to investigate the main physical-chemical characteristics of 3 types of milk: a) cow milk, b) buffalo milk, and c) experimental milk; the experimental milk is obtained from an innovative breeding technique (briefly, a better and wider paddock where to host buffaloes, and a diet based on green forage). The milk samples were taken in the morning and were analysed in triplicate by means of IR spectroscopy (Milkoscan 139, Foss Electric, Hillerød, DK). The values ​​obtained from the samples were compared to the reference values from the INRAN food composition tables (National Research Institute for Food and Nutrition). Moreover, energy-corrected milk (ECM) was used to put all cows on an equal basis for comparative purposes over time: ECM (= 740 kcal) was calculated by using the formula commonly used for buffalo cows 50.

### 4.2. Patients recruitment

Participants were aged between 18 and 75yo, with an average age of 43.2±1.4yo. The subjects enrolled were all with a moderate to high cardiovascular risk, and with the body mass index (BMI) > 25.1 kg/m2.

Moreover, 1 or more of the following criteria were required to make such patients eligible.

Inclusion criteria:

- Body circumference: >88 cm (women) or >102 cm (men);

- Circulating levels of total cholesterol: >190 mg/dl (measured no more than 3 months before the start of the study);

- Circulating levels of LDL cholesterol: >115 mg/dl (measured no more than 3 months before the start of the study);

- Circulating levels of triglycerides: >150 mg/dl (measured no more than 3 months before the start of the study);

- Circulating levels of fasting blood glucose: >110 mg/dl and <126 mg/dl (measured no more than 3 months before the start of the study);

- Hypertension: systolic blood pressure ≥140 or diastolic ≥90 mmHg.

On the other hand, the following exclusion criteria were considered during the patients' selection:

- Fasting triglycerides ≥400 mg / dl;

- Fasting cholesterol> 270 mg / dl;

- Cardiovascular events in the 6 months prior to the study;

- Type-2 Diabetes;

- Renal and/or hepatic insufficiency (creatinine> 1.7 mg / dl and ALT / AST> 2 times the normal values, respectively);

- Anemia (Hb <12 g / dl);

- Anti-inflammatory drugs administered in the last 7days;

- Pregnancy;

- Breastfeeding.

To reduce the operators' bias of the study, the authors carried out a single-blind, randomized, controlled, clinical study. Following the first stage of recruitment, the patients were randomized into two arms:

1) treated group(N=11), and

2) control group(N=9).

Randomization has been obtained through a software able to generate random numbers, then linked to the number of patients' clinical chart. All subjects underwent a nutritional visit, taking into consideration also their clinical history and the baseline of their physiological parameters. The complete lipidic profile (total cholesterol, LDL cholesterol, HDL cholesterol, triglycerides) and glycaemia were investigated on blood samples collected and processed at the analysis lab of Marrelli Hospital (Marrelli Health group, Crotone, Italy). Measurements of intracellular oxidative stress have been performed by using OxiSelect™ Intracellular ROS Assay Kit (Cell Biolabs, Inc.)

### 4.3. Blood sampling and analysis

Blood samples have been collected in EDTA-coated tubes. Glycemia has been measured within 60 minutes by using the Trinder test, a diagnostic test used in medicine to determine the presence of glucose or glucose oxidase.

The dosage of total cholesterol and triglycerides was also performed through the enzymatic-colorimetric Trinder test.

### 4.4. Intracellular oxidative stress assay

Intracellular oxidative stress was measured using the OxiSelect ™ Intracellular ROS Assay Kit (Cell Biolabs, Inc.); this is a cellular test for measuring the activity of hydroxyl, peroxyl, or other reactive oxygen species within a cell (from a salivary sample). Before its use, the salivary sample must be frozen as soon as it is collected; then it is thawed, and finally centrifuged at 4000g for 15 minutes (centrifugation allows to eliminate the excess proteins that interfere with the final reading). The assay uses the 2 'cell permeable fluorogenic probe, 7'-dichlorodihydrofluorescin diacetate (DCFH-DA).

### 4.5. Experimental diet

The experimental diet was administered to patients for 3 months, typically divided into 3 main time-points:

- Time T_0_ - baseline
- Time T_1_ - Intermediate follow-up (1.5 months after T_0_ - only clinical follow up)
- Time T_2_ - Final follow-up and results (3 months after T_0_)

After the preliminary enrolment, patients were asked to follow their own typical diet, also including buffalo milk and its dairy products (treated group), or bovine milk and its dairy products (control group), administered as follows:

- treated group:

200 ml of buffalo milk (daily)

125 ml of buffalo yoghurt (daily)

125 g of buffalo mozzarella (once a week)

125 g of buffalo ricotta (twice a week)

- control group:

200 ml of bovine milk (every day)

125 ml of bovine yoghurt (every day)

125 g of bovine mozzarella (once a week)

125 g of bovine ricotta (twice a week)

### 4.6. Value chain of the experimental foods

The value chain of the experimental foods administered in this study to the patients randomized in the treated group (mozzarella, yogurt and ricotta) are produced with an innovative method developed in the project “Razionale”, currently under investigation in the project “Capsule” on different experimental models. The development of innovative solutions able to create optimal conditions for the buffalos, such as housing space higher than the traditional farm, and a diet rich in fresh green, has led to an increase in functional molecules (such as glycine betaine, GABA betaine, carnitine, acetyl-carnitine, propionyl-carnitine, butyryl-carnitine) in buffalos' milk, meat and related products. The experimental conditions were developed by the company “Le Verdi Praterie” jointly with University of Naples Federico II, and University of Campania Luigi Vanvitelli.

### 4.7. Statistical analysis

Statistical analysis was performed using SPSS (28.0) for Windows 10 (SPSS Inc., Chicago, IL). Differences among groups were evaluated by analysis of variance (ANOVA). A general linear model (multivariate) was used to assess differences between treated and control groups and within them. Results are expressed as mean ± standard error, unless otherwise stated. A statistically significance was accepted at p < 0.05.

Percent variance of weight, body mass index, total cholesterol, LDL, HDL, triglycerides and glycemia between first (T_0_) and (T_2_) second experimental stage was calculated as follows:

-100 - (T_2_/T_0_ * 100).

Where T_0_ and T_2_ are the first and the second measurement of the parameters investigated. T student test was used to compare percent variance of parameters between the two study groups. Statistical significance was set at p < 0.05.

## 5. Conclusions

The presence of many bioactive components in buffalo milk such as carnitine and some modified carnitines, GABA-betaine, creatine, phosphocholine and glycerol-phosphocholine, all essential components for the optimal activity of many organs, gives the buffalo milk and its derivatives physiological versatility and great scientific interest. In fact, many milk peptides play a critical role in regulating oxidative metabolism, essential for cell survival; they are also responsible for activating the pathological mechanisms of atherosclerosis, alterations of metabolism and the development of neoplasms related to oxidative damage to DNA. The content of omega-3 and -6 also make buffalo milk a useful anti-inflammatory defense: their balanced presence in the diet plays an important role in the prevention of heart and metabolic diseases. Through an *in vivo* approach on selected groups of patients with cardio-vascular and dysmetabolic diseases, the health-promoting potential of functional foods of buffalo origin was assessed, achieving good results: decrease in lipemic indexes, decrease in fat mass and reduction in concentrations of nitrite in the collection of saliva. The results obtained, in accordance with the results reported by other studies in the literature, may suggest that a diet based on buffalo-derived functional foods is a good strategy to protect large populations from the most common systemic diseases, as these functional foods, enriched with functional peptides, have clinical impact. We have here reported how such products have reliable anti-hypertensive effects in a very short time. Nevertheless, the current literature endorses these molecules as anti-cholesterolemic, antithrombotic, antioxidant and immunomodulatory players, with biological properties that are going to be even more considered in several medical fields but nevertheless some further studies are obligatory in order to confirm/or not results from this preliminary study. The results carried out from this RCT could be a starting point to promote foods offering high levels of desirable nutrients, to offer several simultaneous health benefits beyond its nutritional value. Future applications could be related to the precision medicine and the forthcoming role of the diet in healthcare protocols; in fact, food we eat naturally contains thousands of bioactive molecules, some of which are similar to bioactive drugs. Precision medicine, coupled to the modern machine learning techniques, can discover such components and help design nutrition that will let us live longer and healthier.

## Figures and Tables

**Table 1:** Nutritional parameters of the bovine milk, buffalo milk, and experimental milk investigated in this study.

	Bovine milk	Buffalo milk	Experimental milk
**Energy value (kcal) / 100g**	64	114	116
**Total proteins g/100g**	3,3	4,5	4,55
**Total fats g/100g**	3,7	9	8,57
**Saturated fats g/100g**	2,4	4,2	4,93
**Total carbohydrates g/100g**	4,9	5,1	5,19
**Lactose g/100g**	4,5	5	4,55
**Mineral salts g/100g**	0,8	0,8	0,9

**Table 2:** Nutritional parameters of bovine mozzarella, buffalo mozzarella, and experimental buffalo mozzarella.

	Bovine Mozzarella	Buffalo Mozzarella	Experimental Mozzarella
**Energy value (kcal) / 100g**	225	272	270,28
**Total proteins g/100g**	18,70	16,70	13,50
**Total fats g/100g**	19,50	24,4	23,67
**Saturated fats g/100g**	15	16	14,60
**Total carbohydrates g/100g**	1	0,70	0,80
**Lactose g/100g**	1	0,40	0,80
**Mineral salts g/100g**	0,60	0,70	0,95

**Table 3:** Main outcomes from clinical and hematological examinations, at the two experimental times (T_0_ and T_2_).

Group	Time	Glycemia (mg/dl)	Blood pressure Max. (mm/Hg)	Blood pressure Min. (mm/Hg)
Treated	T_0_	87.12 ± 3.84	121.15 ± 3.19	77.69 ± 1.60
	T_2_	86.45 ± 3.87	116.82 ± 4.82	74.09 ± 2.47
Control	T_0_	90.67 ± 4.62	131.11 ± 3.83	76.11 ± 1.92
	T_2_	92.11 ± 3.31	137.22 ± 3.38	81.67 ± 2.87

**Table 4:** Percent variation (100 - (T2/T0 * 100)) of weight, body mass index B.M.I., total cholesterol, LDL, HDL, triglycerides and glycemia between first (T_0_) and (T_2_) second time-point.

Group	Variation (%)Weight	Variation (%) B.M.I.	Variation (%)Cholesterol	Variation (%) HDL	Variation (%) LDL	Variation (%)Triglicerids	Variation (%)Glycemia
Control	1,78 ± 11,00	18,70 ± 3,55	0,89 ± 0,96	-8,23 ± 9,67	3,98 ± 2,99	4,99 ± 3,06	-1,56 ± 1,62
Treated	4,20 ± 1,54	4,13 ± 1,77	1,61 ± 3,02	7,41 ± 2,23	1,19 ± 5,11	-10,23± 12,21	-3,66 ± 3,23

## References

[1] D'Onofrio N, Mele L, Martino E, Salzano A, Restucci B, Cautela D, Tatullo M, Balestrieri ML, Campanile G (2020). Synergistic Effect of Dietary Betaines on SIRT1-Mediated Apoptosis in Human Oral Squamous Cell Carcinoma Cal 27. Cancers (Basel).

[2] Salzano A, Neglia G, D'Onofrio N, Balestrieri ML, Limone A, Cotticelli A, Marrone R, Anastasio A, D'Occhio MJ, Campanile G (2021). Green feed increases antioxidant and antineoplastic activity of buffalo milk: A globally significant livestock. Food Chem.

[3] Khan IT, Bule M, Ullah R, Nadeem M, Asif S, Niaz K (2019). The antioxidant components of milk and their role in processing, ripening, and storage: Functional food. Vet World.

[4] Shlisky J, Bloom DE, Beaudreault AR, Tucker KL, Keller HH, Freund-Levi Y, Fielding RA, Cheng FW, Jensen GL, Wu D, Meydani SN (2017). Nutritional Considerations for Healthy Aging and Reduction in Age-Related Chronic Disease. Adv Nutr.

[5] Madhusudan Nayak C, Ramachandra CT, Mahesh Kumar G (2020). A Comprehensive Review on Composition of Donkey Milk in Comparison to Human, Cow, Buffalo, Sheep, Goat, Camel and Horse Milk. Mysore J Agric Sci.

[6] Han X, Lee FL, Zhang L, Guo MR (2012). Chemical composition of water buffalo milk and its low-fat symbiotic yogurt development. Funct food health dis.

[7] Nieves JW (2013). Skeletal effects of nutrients and nutraceuticals, beyond calcium and vitamin D. Osteoporos Int.

[8] Romano R, Giordano A, Chianese L, Addeo F, Spagna Musso S (2011). Triacylglycerols, fatty acids and conjugated linoleic acids in Italian Mozzarella di Bufala Campana cheese. J Food Compos Anal.

[9] Shaikh SR, Edidin M (2007). Immunosuppressive effects of polyunsaturated fatty acids on antigen presentation by human leukocyte antigen class I molecules. J Lipid Res.

[10] Divya KB, Sathish MHK, Kapila S, Sabikhi L (2015). Immunosuppressive Potential of Low Fat Buffalo Milk Supplemented with Omega-3 Fatty Acids. Food Agr Immunol.

[11] Tatullo M, Codispoti B, Paduano F, Nuzzolese M, Makeeva I (2019). Strategic Tools in Regenerative and Translational Dentistry. Int J Mol Sci.

[12] Ahmad S, Anjum FM, Huma N, Sameen A, Zahoor T (2014). Composition and physico-chemical characteristics of buffalo milk with particular emphasis on lipids, proteins, minerals, enzymes and vitamins. J Anim Plant Sci.

[13] Kapadiya DB, Prajapati DB, Jain AK, Mehta BM, Darji VB, Aparnathi K (2017). D. Comparison of Surti goat milk with cow and buffalo milk for physicochemical characteristics, selected processing-related parameters and activity of selected enzymes. Vet World.

[14] Reddi S, Shanmugam VP, Kapila S, Kapila R (2016). Identification of buffalo casein-derived bioactive peptides with osteoblast proliferation activity. Eur Food Res Technol.

[15] Bressan E, Ferroni L, Gardin C, Bellin G, Sbricoli L, Sivolella S, Brunello G, Schwartz-Arad D, Mijiritsky E, Penarrocha M, Penarrocha D, Taccioli C, Tatullo M, Piattelli A, Zavan B (2019). Metal Nanoparticles Released from Dental Implant Surfaces: Potential Contribution to Chronic Inflammation and Peri-Implant Bone Loss. Materials (Basel).

[16] Servillo L, D'Onofrio N, Giovane A, Casale R, Cautela D, Castaldo D, Iannaccone F, Neglia G, Campanile G, Balestrieri ML (2018). Ruminant meat and milk contain δ-valerobetaine, another precursor of trimethylamine N-oxide (TMAO) like γ-butyrobetaine. Food Chem.

[17] Servillo L, D'Onofrio N, Neglia G, Casale R, Cautela D, Marrelli M, Limone A, Campanile G, Balestrieri ML (2018). Carnitine precursors and short-chain acylcarnitines in water buffalo milk. J Agric Food Chem.

[18] Cholewa JM, Guimarães-Ferreira L, Zanchi NE (2014). Effects of betaine on performance and body composition: a review of recent findings and potential mechanisms. Amino Acids.

[19] Cholewa JM, Wyszczelska-Rokiel M, Glowacki R (2013). Effects of betaine on body composition, performance, and homocysteine thiolactone. J Int Soc Sports Nutr.

[20] Atkinson W, Slow S, Elmslie J, Lever M, Chambers ST, George PM (2009). Dietary and supplementary betaine: Effects on betaine and homocysteine concentrations in males. Nutr Metab Cardiovasc Dis.

[21] Kauppinen A, Suuronen T, Ojala J, Kaarniranta K, Salminen A (2013). Antagonistic crosstalk between NF-κB and SIRT1 in the regulation of inflammation and metabolic disorders. Cell Signal.

[22] D'Onofrio N, Balestrieri A, Neglia G, Monaco A, Tatullo M, Casale R, Limone A, Balestrieri ML, Campanile G (2019). Antioxidant and anti-inflammatory activities of buffalo milk δ-valerobetaine. J Agric Food Chem.

[23] D'Onofrio N, Servillo L, Balestrieri ML (2018). SIRT1 and SIRT6 signalings in cardiovascular disease protection. Antioxid Redox Signal.

[24] Singh CK, Chhabra G, Ndiaye MA, Garcia-Peterson LM, Mack NJ, Ahmad N (2018). The Role of Sirtuins in Antioxidant and Redox Signaling. Antioxid Redox Signal.

[25] Perniconi B, Coletti D, Aulino P, Costa A, Aprile P, Santacroce L, Chiaravalloti E, Coquelin L, Chevallier N, Teodori L, Adamo S, Marrelli M, Tatullo M (2014). Muscle acellular scaffold as a biomaterial: effects on C2C12 cell differentiation and interaction with the murine host environment. Front Physiol.

[26] Servillo L, Giovane A, Cautela D, Castaldo D, Balestrieri ML (2014). Where does N(ε)-trimethyllysine for the carnitine biosynthesis in mammals come from?. PLoS One.

[27] Garau V, Manis C, Scano P, Caboni P (2021). Compositional Characteristics of Mediterranean Buffalo Milk and Whey. Dairy.

[28] Basilicata MG, Pepe G, Sommella E, Ostacolo C, Manfra M, Sosto G, Pagano G, Novellino E, Campiglia P (2018). Peptidome profiles and bioactivity elucidation of buffalo-milk dairy products after gastrointestinal digestion. Food Res Int.

[29] Codispoti B, Marrelli M, Paduano F, Tatullo M (2018). NANOmetric BIO-Banked MSC-Derived Exosome (NANOBIOME) as a Novel Approach to Regenerative Medicine. J Clin Med.

[30] Lemosquet S, Delamaire E, Lapierre H, Blum JW, Peyraud JL (2009). Effects of glucose, propionic acid, and nonessential amino acids on glucose metabolism and milk yield in Holstein dairy cows. J Dairy Sci.

[31] Spagnuolo G, Codispoti B, Marrelli M, Rengo C, Rengo S, Tatullo M (2018). Commitment of Oral-Derived Stem Cells in Dental and Maxillofacial Applications. Dent J (Basel).

[32] Tatullo M, Marrelli B, Zullo MJ, Codispoti B, Paduano F, Benincasa C, Fortunato F, Scacco S, Zavan B, Cocco T (2020). Exosomes from Human Periapical Cyst-MSCs: Theranostic Application in Parkinson's Disease. Int J Med Sci.

[33] Salzano A, Licitra F, D'Onofrio N, Balestrieri ML, Limone A, Campanile G, D'Occhio MJ, Neglia G (2019). Short communication: Space allocation in intensive Mediterranean buffalo production influences the profile of functional biomolecules in milk and dairy products. J Dairy Sci.

[34] Proestos C (2018). Superfoods: Recent Data on their Role in the Prevention of Diseases. Curr Res Nutr Food Sci.

[35] Signorini L, Ballini A, Arrigoni R, De Leonardis F, Saini R, Cantore S, De Vito D, Coscia MF, Dipalma G, Santacroce L, Inchingolo F (2021). Evaluation of a Nutraceutical Product with Probiotics, Vitamin D, Plus Banaba Leaf Extracts (Lagerstroemia speciosa) in Glycemic Control. Endocr Metab Immune Disord Drug Targets.

[36] Penchev P I, Ilieva Y, Ivanova T, Kalev R (2016). Fatty acid composition of buffalo and bovine milk as affected by roughage source-silage versus hay. Emir J Food Agric.

[37] Tatullo M, Genovese F, Aiello E, Amantea M, Makeeva I, Zavan B, Rengo S, Fortunato L (2019). Phosphorene Is the New Graphene in Biomedical Applications. Materials (Basel).

[38] Khan IT, Nadeem M, Imran M, Ullah R, Ajmal M, Jaspal MH (2019). Antioxidant properties of Milk and dairy products: a comprehensive review of the current knowledge. Lipids Health Dis.

[39] Pisano MB, Scano P, Murgia A, Cosentino S, Caboni P (2016). Metabolomics and microbiological profile of Italian mozzarella cheese produced with buffalo and cow milk. Food Chem.

[40] Salzano A, Manganiello G, Neglia G, Vinale F, De Nicola D, D'occhio M, Campanile G (2020). A preliminary study on metabolome profiles of buffalo milk and corresponding mozzarella cheese: safeguarding the authenticity and traceability of protected status buffalo dairy products. Molecules.

[41] Abd El-Salam M, El-Shibiny (2011). A comprehensive review on the composition and properties of buffalo milk. Dairy Sci Technol.

[42] De Vito D, Monno R, Nuccio F, Legretto M, Oliva M, Coscia MF, Dionisi AM, Calia C, Capolongo C, Pazzani C (2015). Diffusion and persistence of multidrug resistant Salmonella Typhimurium strains phage type DT120 in southern Italy. Biomed Res Int.

[43] Bonadies I, Di Cristo F, Valentino A, Peluso G, Calarco A, Di Salle A (2020). pH-Responsive Resveratrol-Loaded Electrospun Membranes for the Prevention of Implant-Associated Infections. Nanomaterials (Basel).

[44] Marrelli M, Tatullo M, Dipalma G, Inchingolo F (2012). Oral infection by Staphylococcus aureus in patients affected by White Sponge Nevus: a description of two cases occurred in the same family. Int J Med Sci.

[45] Woodworth HL, McCaskey SJ, Duriancik DM, Clinthorne JF, Langohr IM, Gardner EM, Fenton JI (2010). Dietary fish oil alters T lymphocyte cell populations and exacerbates disease in a mouse model of inflammatory colitis. Cancer Res.

[46] Perić J, Drinić M, Mićić N (2019). Fatty acids in feed of laying hens on the production parameters and the ratio of omega-6 and omega-3 fatty acids. J Anim Sci Biotechnol.

[47] Simopoulos AP (1999). Essential fatty acids in health and chronic disease. Am J Clin Nutr.

[48] D'Onofrio N, Cacciola NA, Martino E, Borrelli F, Fiorino F, Lombardi A, Neglia G, Balestrieri ML, Campanile G (2020). ROS-Mediated Apoptotic Cell Death of Human Colon Cancer LoVo Cells by Milk δ-Valerobetaine. Sci Rep.

[49] Fusco A, Dicuonzo G, Dell'Atti V, Tatullo M (2020). Blockchain in Healthcare: Insights on COVID-19. Int J Environ Res Public Health.

[50] Campanile G, De Filippo C, Di Palo R, Taccone W, Zicarelli L (1998). Influence of dietary protein on urea levels in blood and milk of buffalo cows. Livestock Production Science.

